# La Charite

**Published:** 1831

**Authors:** 


### LA CHARITE.

### Clinique of Messrs. Boyer and Roux.

From the multitude of cases without interest, facts without value, and reflections without judgement, published in the French journals, the wearied eye turns to settle with delight on a "clinique d'hôpital." In this we find less of that imaginative feeling which distinguishes our Gallic brethren, and the spirituality of the nation appears to succumb under the influence of experience and of common sense. The cases detailed by private practitioners in France are, for the most part, utterly valueless. In that land of liberty and equality, of spiritualism and republicanism, knowledge, or at all events professional knowledge, is monopolized to a degree unknown in this island of monopolies. The Parisian, and the hospital surgeons in the great provincial towns, possess all the current information, and though they naturally share in the spirit of vague generalization and eager disposition to refine which characterise their nation, yet the facts which present themselves before them tend to sober their theories, and dash their speculations with some of that caution and deliberation which their English compeers are more prone to exercise. On this account it is, that the hospital reports, and the productions of hospital surgeons and physicians, are almost exclusively deserving of attention in the French periodicals. We need not say that with us this is far from true. We shall proceed to notice a case or two from the clinique of Messrs. Boyer and Roux.

*Fungous Tumour of the Radius—Ligature of the Brachial Artery—Amputation—Death.**

On the 5th November, 1830, a man, æt. 35, was admitted into La Charité, with a tumour, nearly the size of the fist, on the inferior and anterior part of

---

* Hebdomadaire, T. iv. No. 40.

the fore-arm. The radial artery was thrown forwards by the tumour, which evidently increased in size when the arm was dependent. Obscure pulsation was distinguished in the tumour, which, when pressed, crackled like a snow-ball, and communicated to the fingers the sensation of a bony shell. The patient referred the origin of his disease to the preceding May. In squeezing a sponge he felt a crack at the lower end of the radius, soon afterwards an irregular swelling with redness of the skin, and then a distinct tumour which was punctured, but gave issue only to blood. From the symptoms past and present, it was imagined that the tumour was one of fungus hæmatodes, (fongueuse sanguine,) and that it had its origin in the cancellar structure of the radius. As pressure on the brachial artery arrested the pulsation in it, it was determined to tie that vessel, which was done on the 7th December. The pulsation in the tumour ceased; the tumour itself diminished in volume, and became hard; ice was applied, and subsequently compresses dipped in a solution of tannin with a bandage; the ligature came away from the artery on the 25th day; and appearances were so far favourable. But as soon as the patient began to use the limb the disease returned with rapidity, and assumed a more malignant aspect than before. Amputation was performed in the upper third of the fore-arm, where the parts were sound. The patient died of the effects of the amputation, (what they were is not stated); but nothing of consequence was discovered on opening the body. On examining the amputated limb, there was found beneath the skin and the extensor muscles, which were separated from each other, a fleshy mass surrounding the inferior extremity of the radius. The external layer of the tumour was of lardaceous consistence, in the centre it was softened, cerebriform, with clots of blood. No trace of the radius was found in the centre of the tumour, the bone being merely connected with its external layers above. Below, the tumour was separated from the articulation of the wrist by a layer of cartilage, apparently that belonging to the lower end of the radius.

This appears to have been a genuine case of fungus hæmatodes. The improvement after the ligature of the brachial artery, followed by local pressure, though singular, is not very surprising to those who have seen many cases of the disease.

Case 2. *Hydrocele and Hæmatocele—Puncture and Injection—Incision—Cure.*

A man was affected with a hydrocele of moderate size, when, after a contusion of the part, its volume suddenly augmented. After a while the tumefaction became softer, but there was no transparency, and the weight was greater than that of an ordinary hydrocele. M. Roux now made a puncture in the upper part of the tunica vaginalis, when a quantity of blackish fluid escaped through the canula, and the tumour had almost disappeared. M. Roux now injected some warm water, inflammation followed, and fluctuation became evident at the point where the puncture had been made. The tunica vaginalis was now laid freely open by an incision, the wound dressed in with charpie, suppuration and granulation freely established, and the patient ultimately cured. We need scarcely say that this practice has the sanction of the most experienced surgeons in this country. Hæmatocele, to any extent, is best treated by incision of the tunica vaginalis.

Case 3. *Lithotomy for the Extraction of a Portion of Gum Catheter in the Bladder.*

A man, ætatis 67, was admitted into La Charité, labouring under the symptoms of retention of urine. The patient was affected with paralysis of the bladder, and on the preceding night a surgeon in the town had drawn off the urine by means of a gum catheter, and left the catheter in the bladder and urethra. The instrument had retired within the urethra, and its point lay buried behind the corona glandis, when ineffectual attempts were made to extract it, and it passed in still more deeply than before, and lay altogether in the bladder! On the patient's admission into the hospital the catheter was readily introduced, and a great quantity of urine drawn off. The foreign body was distinctly felt, and an immediate operation was urged upon the patient. On the next day the lateral operation was performed. On introducing the forceps the catheter could not be seized, when M. Roux passed his finger into the bladder, drew down the catheter to its neck, and extracted it with a pair of polypus forceps; the catheter was ten inches in length. A gum catheter was retained in the urethra after the operation. The patient died in the course of a week of "a febrile affection with general depression." No particulars of the post-mortem inspection are afforded.

The reporter of the case passes some strictures on the conduct of the surgeon who allowed the catheter to slip into the bladder. Having found it in the urethra, he might, at all events, have prevented it from going farther, by the simple expedient of squeezing the penis upon the bougie at the pubes. It is difficult to imagine how a gum catheter could pass entirely into the bladder, indeed we doubt if it would do so, unless thrust into it by injudicious and clumsy attempts at its extraction from the urethra. For further information respecting the presence of portions of bougie or other foreign bodies in the bladder, the reporter refers the inquisitive reader to Chopart. Some little time back we saw a hair-pin extracted from the male perineum. There was no external wound, and how it got there the patient professed himself totally unable to conceive. No bad consequences followed its extraction by an incision in perineo.

---

